# Medical and Philosophical Intelligence

**Published:** 1813-12

**Authors:** 


### MEDICAL AND PHILOSOPHICAL INTELLIGENCE.

### IMPERIAL INSTITUTE OF FRANCE.

### *Transactions of the Imperial Institute of France for* 1812.

SPALLANZANI, in a very celebrated work, having applied the gastric juice out of the stomach to every kind of food, affirmed that it produced, when assisted by heat, effects nearly similar to those produced in the stomach itself. This philosopher went so far as to ascribe to this gastric juice, thus separated, the property of stopping putrefaction. He drew this conclusion from his observations, which has been tacitly adopted by most physiologists, that the gastric juice produces its effects in consequence of its peculiar nature, of its composition, and affinities.

M. de Montegre, Doctor of Medicine, having the power of throwing up, without inconvenience, what he has in his stomach, has thought of employing this power in order to determine the different points of the received doctrine respecting digestion. When he throws up the contents of his stomach while fasting, he obtains a notable quantity of a liquid which he considers as true gastric juice, and which he examined with respect to its chemical properties as well as its action on the food.

He found this liquid resemble saliva; but its action appeared to him very different from the statement of Spallanzani. When exposed to a temperature similar to that of the human body, in phials placed under the arm-pit, it putrefied exactly like saliva. It did not stop the course of putrefaction in other substances, except when it was acid; and by adding a little vinegar to saliva it was made to possess the same property. This acidity is not essential; and when M. de Montegre swallowed enough of magnesia to absorb it, the digestion went on as well as usual. Acidity appeared again in a little time: even when M. de Montegre mixed the food which he swallowed with magnesia, it became acid after a sufficient time.

These experiments, repeated a great number of times, and with all the requisite precautions, have induced the author to conclude that the gastric juice does not differ from saliva, that it cannot stop putrefaction, nor produce digestion independent of the vital action of the stomach; and that the acidity which appears, and which the food evolves during digestion, is an effect of the action of the stomach.

It is much to be wished that M. de Montegre would continue his researches, and make them also upon those animals that Spallanzani employed, that we may determine what to think of a doctrine which has for a considerable time been generally embraced.

M. de Blainville, Joint Professor to the Faculty of Sciences of Paris, has described at full length the forms of articulation of the fore-arm and arm in different animals, and determined the motions which each of these forms makes necessary, chiefly with regard to the greater or smaller facility of rotation. This dissertation, on a point of importance portance relative to the mechanism of animals, is interesting also as far as regards their classification; for the degree of rotation of the fore-arm having considerable influence on the address of the animals, ought to be considered, as far as regards the degrees of perfection, and of course influences their natural affinities.

The same anatomist has presented a memoir on the form of the sternum in birds. As this bone, or rather this great bony surface, resulting (as M. Geoffroy has shown) from the union of five different bones, gives origin to the principal muscles of the bird, the more solid and extended it is, the more solid a point of support does it furnish to these muscles, and the more ought it to contribute to render the flight powerful. It ought, therefore, to have an influence over the whole economy of the bird, and give useful indications respecting the classification of these animals. M. de Blainville draws his indications from the membranous spaces, more or less extended, which supply the place of bone in a part of the sternum. He adds the consideration of the fork, and of some organs connected with it, and in most cases finds a great agreement between the disposition of these parts and the natural families. However, there exist exceptions so manifest, that we cannot entirely confide in this new way of classification.

M. Marcel de Serres, Professor to the Faculty of Sciences of Montpellier, has drawn up a laborious work on the anatomy of insects, and particularly on their intestinal canal, which he has described with much detail in a great number of species. His object was to determine the functions peculiar to the different parts of the canal and its appendages: and, besides his dissections, he has made ingenious experiments on living individuals. Colored liquors injected into the cavity of the peritonæum were absorbed by long slender vessels, which always adhere to some part of the intestinal canal: hence he conceives that the use of these vessels is to secrete from the common mass of humors digestive liquors, and to throw them into the canal. An attentive examination of certain sacks, which in some genera have been considered as stomachs, in others as cœcums, and the certainty acquired that the food does not enter there, but on the contrary that they are found full of bilious liquor, has induced M. de Serres to conclude that they are reservoirs of that humor.

He deprives the grasshoppers, and the analogous genera, of the quality of ruminating animals, which had been ascribed to them, and he has convinced himself that these animals do not bring the food back to the mouth; but that they throw out only in certain circumstances this biliary juice, of which they have so great a quantity. This memoir contains many other curious observations on the form of the intestinal canal, the proportions of its parts, and their relation to the disposition of insects.

M. Dutrochet, physician at Chateau-Renaud, department of the Indre, has made a curious observation on the gestation of the viper. He assures us that the young vipers have their umbilical vessels distributed not only on the yoke of the egg, in which they are at first enclosed, but that a part of these vessels is distributed likewise on the the internal surface of the oviducts, and forms a net which may be considered as a real placenta. The vipers in that case would participate in the mode of nutrition peculiar to the mammalia, and in that hitherto conceived to belong exclusively to the whole class of oviparous animals.

*Medicine and Surgery.*—After twelve years of experiments, made in every civilized country, since the discovery of vaccination, the Class conceived that it would be useful to collect the result of the observations on an object so important to humanity. Another motive rendered this undertaking necessary: objections and doubts had been raised by well-informed men, whose testimony was calculated to influence public opinion. It has even been questioned, whether small-pox inoculation, considered as a preservative, and in some cases as a remedy for various diseases, was not preferable to vaccination, or at least entitled to be preserved as well as it.

MM. Berthollet, Percy, and Hallé, commissioners, undertook the necessary researches to satisfy the intention of the Society; and presented, by means of M. Hallé, a long report, which the Class ordered to be printed. They bring the different points of discussion to six principal questions. Under these different heads they unite, as far as possible, every thing that has been accurately ascertained respecting the effects of vaccination in Europe, and in the countries where Europeans have been able to introduce vaccination.

They have collected a great many facts, observed particularly in France, England, Italy, India, and America, and observed in individuals of all classes, constitution, mode of life, habits, and manners, exceedingly different from each other. On the other hand, they endeavor to estimate the value of the principal facts upon which have been founded the most plausible objections, which they neither attempt to elude nor conceal. Thus, by comparing together the observations, they have been led to the conclusions with which they terminate their report: namely—

That vaccination does not introduce into the body a matter capable of producing a remarkable disturbance, and which requires to be expelled by a movement similar to that which results from inoculation: that the eruptions which sometimes appeared at first were not owing to the cow-pox matter; but to other circumstances, in the midst of which these vaccinations were performed:

That the unfortunate results which sometimes occurred were owing to causes altogether foreign, which made their appearance during the course of vaccination, and owing entirely to the state of the patients:

That the disorders following vaccination, when not owing to pre-existing diseases, have been very particular cases, depending upon circumstances peculiar to individuals; and that their number bearing no proportion to the immense number of observations exempt from accidents of any kind, no general consequence can be drawn from them:

That the unfortunate results, even supposing them incontestible, are more than compensated by the numerous instances of chronical and and obstinate diseases which have been completely removed by vaccination; examples which, when compared with similar effects from inoculation, especially if we take into consideration the greater danger of inoculation, leave the superiority greatly on the side of vaccination:

Finally, that the preservative virtue of vaccination, when the virus has been properly taken, and the pock has proceeded properly, is fully as great as that of small-pox inoculation; while it possesses the immense advantage of circumscribing small-pox epidemics, and affords reasonable hopes of finally annihilating this dreadful scourge of humanity.

M. Portal has published a new edition of his treatise on asphyxias, a work printed and circulated by order of government, for the instruction of the people, and which has probably saved the lives of thousands of citizens, since it has been circulated in France, and in all the rest of Europe, by the numerous translations that have been made of it.

M. Dumas, correspondent and dean of the Faculty of Medicine at Montpellier, has published a considerable work, entitled *General Doctrines of Chronical Diseases*, in which he considers this important subject in the most general point of view. Not confining himself to the external forms of these diseases, he ascends to the principles of their phenomena, determining by analysis the simple affections of which they are composed, and which may be considered as their elements. An accurate comparison of acute and chronic diseases induces him to conclude that there is no constant character which separates these two classes of diseases. In his account of chronic diseases, he shows that the want of nutrition and emaciation are produced more speedily by diseases connected with respiration than with the organs of digestion. He shows the constant relation between certain external forms, and a disposition to peculiar chronic diseases. Hence he deduces the character of the physiognomy peculiar to each.

The study of the revolutions natural to these diseases has made him perceive a period within which it is still possible to prevent their formation: different kinds of crises which succeed it, and what may render these crises advantageous or injurious: the different changes of acute into chronic diseases, and *vice versâ*, and the cause of these alternations.

The determination of the simple affections of which these diseases are composed, or of their pathological elements, appeared to him of the greatest importance; since it furnishes us, in some measure, with the means of simplifying them, by attacking these elements one after another, beginning with the most powerful. This fundamental point of view has enabled him to explain their formation, and to determine in a solid manner the method of treating them; but for this purpose it was necessary to draw an accurate line between the essential elementary affections and the symptomatic.

Thus he has risen by degrees to the general phenomena, and has been able to deduce them from a small number of primitive affections.

His

His theory of the formation of chronic diseases reduces itself to the relations of the elementary affections to each other, and to the system of organs which they occupy.

M. Dumas treats, in a manner which he considers as new, every thing that regards the general disposition to chronic diseases. He establishes a difference between the constitution and the temperament, which are sometimes opposed to each other; and this opposition is the most direct cause of a tendency to chronic diseases. He estimates the influence of time of life by the relation between the elementary affections, from which results a disposition at every age to different kinds of diseases, modifications in the diseases common to each age, and changes advantageous or hurtful during the progress of each disease. He treats of the passions after analogous views. Each of them may be decomposed into a certain number of simple affections, which metaphysics knows and enumerates.

Finally, M. Dumas arrives at his last part, which is that of the treatment. He shows the justice of his doctrine, by making it appear that all the approved methods of treatment are easily reducible to the principles which he has established. He finishes with some interesting observations on hereditary and on incurable diseases.

In an appendix, M. Dumas gives several examples of the manner in which he thinks the particular and detailed history of the elementary affections may be drawn up. A second work, which he promises, will establish and explain, by examples drawn from his practice, every thing difficult and abstract which this general doctrine contains.

---

*Society for Relief of Widows and Orphans of Medical Men in London and its Vicinity.*—On Friday, the 29th of October, the twenty-fifth Anniversary Festival of this Society was celebrated at the City of London Tavern, Bishopsgate-street.

The royal patron, his Royal Highness the Duke of Kent, presided; and from the lively interest he took in the concerns of the Society, proved himself more than merely a *nominal* patron to this useful institution.

His Royal Highness took a most accurate view of the Society since its first establishment, and recounted the relief that had been afforded to the widows and orphans down to the present time.

He was happy, he said, to be able to report, that no application from any family whose claim to relief could be by any means substantiated, agreeably to the laws of the Society, had been unattended to; and that those who were now pensioners on the establishment, were satisfied with the sums awarded to them, and grateful to the directors for their liberality.

His Royal Highness at the same time could not help expressing his regret, on finding, by inspection of the lists of physicians, surgeons, and apothecaries, resident in and near the metropolis, that not more than one third of the professors of the different branches of the medical science, were enrolled among the members of an institution whose object was to afford comfort and relief to the widows and children of the less fortunate members of the profession; and expressed a hope that the time would come when it would be considered a disgrace to each regular member of the medical profession, *not* to belong to this Society.

The anniversary dinners of this Society being more calculated for bringing the members of the profession and their friends together to enjoy social conviviality, than for the purpose of soliciting donations, it is not usual to hand a plate about after dinner, nor to solicit contributions from the company present. Whatever donations are given, are unsolicited; and at this meeting they amounted to a considerable sum.

One hundred guineas were handed to the treasurer, being the gift of Sir Frederic Baker; and Sir Henry Halford presented the Society with fifty guineas. Other donations of minor consideration were presented by several of the gentlemen present; and Dr. Lettsom announced a bequest of five hundred pounds to the Society from the late Dr. Anthony Fothergill, of Bath. Several new members were proposed, and the company broke up at a late hour, highly pleased with the transactions of the day.

At the last audit, September 15th, the state of the fund was as follows:—three per cent consols, 20,400*l.*; navy five per cents, 200*l.*

*Deaths by Small-pox, from the Weekly Bills.*

| | | | | | |
|---|---|---|---|---|---|
| Aug. | 10, | 10 | Oct. | 5, | 16 |
| | 17, | 17 | | 12, | 13 |
| | 24, | 10 | | 19, | 16 |
| | 31, | 16 | | 26, | 23 |
| Sept. | 7, | 4 | Nov. | 2, | 25 |
| | 14, | 11 | | 9, | 16 |
| | 21, | 18 | | 16, | 17 |
| | 28, | 12 | | | |

Mr. Calcraft presented a petition, on Friday the 19th of November, to the Honorable the House of Commons, for leave to bring in a Bill "for regulating the Profession and Practice of Apothecaries, Surgeon-apothecaries, and Practitioners in Midwifery, in England and Wales." We are authorised to state, that the General Committee do not mean to introduce the Bill till after the Christmas recess; and that it will be previously printed and published. As many of our readers may not be acquainted with the forms of the House, and much misconception and confusion consequently took place during the progress of the Bill that was last session read the first time, and then withdrawn, we take this opportunity of remarking, that, *after* a Bill has been printed, and read the first time, parties interested may petition for or against it; and that, when it has been read the second time, and ordered by the House to go to a committee, it may then receive such modifications as may be deemed necessary.

Mr. Stevenson, Oculist and Aurist to her Royal Highness the Princess of Wales, will recommence his annual Course of Lectures on the Anatomy, Physiology, and Diseases of the Eye and Ear, about the middle of January.

Dr. Merriman will recommence his Lectures on Midwifery and the Diseases of Women and Children, on Monday, December 6, at half-past ten o'clock.

*Preparation of the Porporino Red, by* LAMPADIUS.—The name of *porporino* is given at Rome to an artificial animal substance, which is employed for engraving in stone and the mosaic work. Different shades of it are found in St. Peter's church, where it is employed as an ornament.

The *porporino* red is a fine brown red, its fracture is scaly, it has a very little polish, and is of considerable weight. This mass fuses in the fire, and is afterwards run into moulds. It is so hard that it can be used in all the operations of engraving on stone.

M. Lampadius having obtained a piece, after several experiments he completely succeeded in his endeavors to imitate it, in the following manner. He took two parts of very white sand, one of pure clay, one and a half of pure minium, half a part of purified potash, half a part of white arsenic, and four parts of saltpetre.

When all these ingredients were well pounded, and mixed in a marble mortar, he added five parts of fine and perfectly pure copper filings, mixing the whole well together.

He afterwards took a Hessian crucible; and making it red in the fire, he put the mixture into it with a ladle, and covered it with a cover made to fit exactly, that none of the fuel might mix with it; he then let the whole remain in fusion for an hour.

In the mean time he heated a clay mould, selected for the purpose, the inside of which was chalked, that the mass might not adhere to it. When the mould was heated to incandescence, the mass was poured into it, covered over with a lid, also heated, and the whole left to cool very slowly; for if it cool suddenly the mass becomes brittle. He was particularly careful to choose the ingredients very pure, not to stir the mass with an iron instrument, to prevent the admission of any dust from the fire, and to use nitre that was entirely free from muriatic acid.

---

*New Example of Combustion during Combination.*—It is well known that when sulphur is made to combine with copper, iron, and some other metals, previously reduced to powder, and well mixed, the compound becomes red hot, and glows like a live coal just at the instant of its formation. The same thing takes place when phosphorus is made to unite with lime, barytes, and strontian. When quicklime is slacked in obscurity, it frequently becomes luminous; and there is little doubt that the same thing would happen to barytes and strontian. Chevreul has lately observed that when barytes or strontian is heated in contact with muriatic acid gas, the gas is absorbed, and the earthy salt formed becomes red hot. There can be little doubt that this evolution of heat is owing to the condensation of the gas. It is true that in the present case the agency of oxygen is not excluded; for if we adopt the opinion of Davy respecting the composition of muriatic acid, it is obvious that in the present case a double decomposition takes place. The chlorine of the muriatic acid unites with the metallic basis of the barytes, and forms *barytane*, while the hydrogen of the muriatic acid combines with the oxygen of the barytes, and forms water; but if we consider that both muriatic acid and barytes are products of combustion, it will be obvious that the presence of the oxygen alone cannot account for the light and heat evolved.

